# Population-Based Prevalence of *Chlamydia trachomatis* Infection and Antibodies in Four Districts with Varying Levels of Trachoma Endemicity in Amhara, Ethiopia

**DOI:** 10.4269/ajtmh.20-0777

**Published:** 2020-10-26

**Authors:** Scott D. Nash, Tigist Astale, Andrew W. Nute, Danaya Bethea, Ambahun Chernet, Eshetu Sata, Mulat Zerihun, Demelash Gessese, Gedefaw Ayenew, Zebene Ayele, Berhanu Melak, Mahteme Haile, Taye Zeru, Zerihun Tadesse, Benjamin F. Arnold, Elizabeth Kelly Callahan, Diana L. Martin

**Affiliations:** 1 Trachoma Control Program, The Carter Center, Atlanta, Georgia;; 2 Trachoma Control Program, The Carter Center, Addis Ababa, Ethiopia;; 3 Centers for Disease Control and Prevention, DPD, Atlanta, Georgia;; 4 Amhara Public Health Institute, Research and Technology Transfer Directorate, Bahir Dar, Ethiopia;; 5 Francis I. Proctor Foundation, University of California San Francisco, San Francisco, California;; 6 Department of Ophthalmology, University of California San Francisco, San Francisco, California

## Abstract

The Trachoma Control Program in Amhara region, Ethiopia, scaled up the surgery, antibiotics, facial cleanliness, and environmental improvement (SAFE) strategy in all districts starting in 2007. Despite these efforts, many districts still require additional years of SAFE. In 2017, four districts were selected for the assessment of antibody responses against *Chlamydia trachomatis* antigens and *C. trachomatis* infection to better understand transmission. Districts with differing endemicity were chosen, whereby one had a previous trachomatous inflammation-follicular (TF) prevalence of ≥ 30% (Andabet), one had a prevalence between 10% and 29.9% (Dera), one had a prevalence between 5% and 10% (Woreta town), and one had a previous TF prevalence of < 5% (Alefa) and had not received antibiotic intervention for 2 years. Survey teams assessed trachoma clinical signs and took conjunctival swabs and dried blood spots (DBS) to measure infection and antibody responses. Trachomatous inflammation-follicular prevalence among children aged 1–9 years was 37.0% (95% CI: 31.1–43.3) for Andabet, 14.7% (95% CI: 10.0–20.5) for Dera, and < 5% for Woreta town and Alefa. *Chlamydia trachomatis* infection was only detected in Andabet (11.3%). Within these districts, 2,195 children provided DBS. The prevalence of antibody responses to the antigen Pgp3 was 36.9% (95% CI: 29.0–45.6%) for Andabet, 11.3% (95% CI: 5.9–20.6%) for Dera, and < 5% for Woreta town and Alefa. Seroconversion rate for Pgp3 in Andabet was 0.094 (95% CI: 0.069–0.128) events per year. In Andabet district, where SAFE implementation has occurred for 11 years, the antibody data support the finding of persistently high levels of trachoma transmission.

## INTRODUCTION

The WHO recommends the surgery, antibiotics, facial cleanliness, and environmental improvement (SAFE) strategy to eliminate trachoma as a public health problem. To monitor the impact of the SAFE strategy, programs rely on population-based surveys to estimate the prevalence of the clinical sign trachomatous inflammation-follicular (TF) measured among children aged 1–9 years. The threshold for elimination of trachoma as a public health problem is < 5% TF among this age-group. Although field-workers participating in trachoma surveys can be trained to grade TF reliably, TF often overestimates the infection prevalence of the causative agent *Chlamydia trachomatis*, particularly in post–mass drug administration (MDA) settings.^1–4^ Other indicators of trachoma, such as trachomatous inflammation-intense (TI), *C. trachomatis* infection measured using a nucleic acid amplification test, or antibody responses to *C. trachomatis* antigens, have in large part been limited to research settings and are not currently used for programmatic decision-making.

Antibody responses to *C. trachomatis* antigens have recently been used to measure the cumulative exposure to the bacterium among trachoma-affected or previously affected populations.^5,6^ In particular, antibodies against the *C. trachomatis* antigens Pgp3 and CT694 have been shown to be present in those infected with *C. trachomatis*, to increase with age in trachoma-endemic populations, and to be at low prevalence in populations receiving MDA with antibiotics.^5,7–10^ However, the programmatic role of these markers in trachoma control is still being determined. More data are needed from a range of settings to better understand the epidemiology of *C. trachomatis*–specific serological markers.

The Trachoma Control Program in Amhara region, Ethiopia, has been at scale with the SAFE strategy since 2007.^11,12^ After 8–11 years of SAFE interventions, not all districts (locally known as woredas) have reached the elimination threshold, and some districts remain with a hyperendemic TF level (≥ 30%).^2,4,12^ Evaluating alternative indicators of *C. trachomatis* infection could help to better understand ocular *C. trachomatis* transmission patterns in districts with persistently high trachoma. In 2017, as part of routine trachoma impact and surveillance surveys conducted in Amhara, dried blood spots (DBS) were collected from a population-based sample of children aged 1–9 years along with ocular swabs collected from children aged 1–5 years in four districts with historically different trachoma endemicity. The aim of this study was to determine the seroprevalence of antibodies to Pgp3 and CT694 and the prevalence infection to better elucidate ocular *C. trachomatis* transmission patterns in districts which are slow in reaching elimination targets.

## METHODS

### Ethics statement.

The study protocol was approved by the Emory University Institutional Review Board (IRB) (protocol 079-2006), the Amhara Regional Health Bureau, and the Federal Ministry of Science and Technology of Ethiopia. Staff from the U.S. CDC did not have contact with study participants or access to identifying information and were determined to be not engaged in research on human subjects. Because of the high illiteracy rate among the population, IRB approval was obtained for oral consent or assent for older children. Oral consent or assent was obtained and recorded electronically for all individual participants according to the principles of the Declaration of Helsinki. Respondents were allowed to terminate the examination at any point without a need of explanation.

### Survey design.

**Figure 1.**
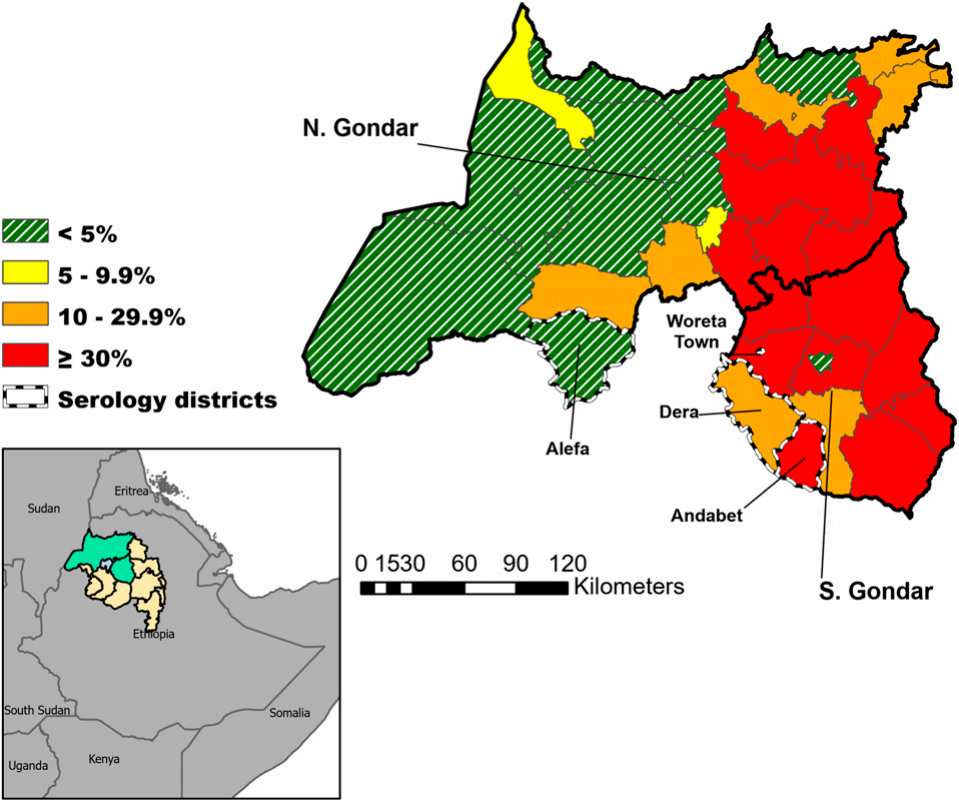
Location of four districts monitored for serological markers of trachoma and corresponding trachomatous inflammation-follicular district-level prevalence, Amhara, Ethiopia, 2017. This figure appears in color at www.ajtmh.org.

Between October and December 2017, DBS and ocular swabs were collected from a population-based sample of children alongside routine trachoma impact and surveillance surveys in four districts in the Amhara region of Ethiopia (Figure 1). One district chosen had a previous TF prevalence of ≥ 30% (Andabet), one had a previous prevalence between 10 and 29.9% (Dera), and one had a prevalence between 5 and 10% (Woreta town) from surveys conducted between 2011 and 2016. A surveillance survey was conducted in the fourth district, which had a previous prevalence of < 5% (Alefa) at an impact survey conducted in 2015. The first three districts had received a community-wide MDA with azithromycin approximately 8 months before the survey, whereas Alefa district had not received MDA for a period of approximately 2.5 years. Mass drug administration coverage as reported from administrative records was consistently high in these districts over 3 years before the surveys (Supplemental Table 1).

As is standard for recently conducted trachoma surveys across Ethiopia, sample size calculations assumed a TF prevalence of 4% ± 2% among children aged 1–9 years, with a design effect of 2.71 and 20% nonresponse, requiring a total of 1,200 children.^13^ Sample size assumptions were set to detect a prevalence below the elimination threshold and were the same regardless of the previous TF prevalence in a district. Following the guidance of the Federal Ministry of Health and the Tropical Data service for trachoma surveys, the survey targeted 30 clusters of 30 households to achieve the desired sample size. Woreta town was a small district (population approximately 41,000), and therefore 20 clusters of 40 households were selected in that district. It was assumed that because seroprevalence is normally larger than TF prevalence for a given district, TF sample size assumptions would be suitable for both indicators. However, for logistical and financial reasons, the serological subset consisted of 22/30 (73.3%) clusters randomly selected in each district for Andabet, Dera, and Alefa districts and 12/20 (60.0%) clusters randomly selected in Woreta town.

A multistage cluster-random survey was conducted in all four districts. In the first stage of sampling, a list of villages (locally called gotts) in each of the districts was created according to geographical distribution within the district. The total number of villages of each district was divided by the total number of villages to be selected to derive the sampling interval. The first village (survey cluster) was selected randomly, and each subsequent cluster was selected by adding the sampling interval to the previous number until 30 clusters were reached (or 20 clusters in the case of Woreta town).

In Ethiopia, each village is composed of existing segments called development teams.^2^ With the help of village leaders, development teams were arranged into segments of approximately 30 households by survey teams (or 40 households for Woreta town), and one segment was randomly selected by the village leaders as the second stage of sampling. All households within each selected segment were surveyed. All individuals aged 1 year and older who resided in the selected households were eligible to participate in the survey. Attempts were made to return to households where household members were absent during the initial visit to examine the absentees.

### Training.

All trachoma graders participated in a training before the survey. To participate in the survey, graders had to pass a slide reliability test of 50 standardized conjunctival images as well as a field-based reliability examination which consisted of grading trachoma among 50 children. The trainees’ scores were compared with the score of a single “Master Grader” certified by the Tropical Data service.^13^ Trainees were required to achieve a minimum kappa score of 0.7 for the sign TF to join field teams for the survey.

### Data collection.

After a household questionnaire, which gathered data on latrine and water presence, was completed by a household representative, all present, consenting individuals aged ≥ 1 year within each household were assessed for the WHO-simplified trachoma signs TF, TI, and trachomatous trichiasis (TT) using a ×2.5-magnification loupe and a flashlight.^14^ Data were entered into cellphones through the Tropical Data application.^13^ Individuals identified as having TF or TI were offered tetracycline eye ointment and directions for use, and individuals identified as having TT were encouraged to participate in the next available TT surgical campaign.

### *Chlamydia trachomatis* infection.

Before the start of fieldwork, eight of the survey clusters (non-serology clusters) were randomly chosen for conjunctival swabbing to measure the presence of *C. trachomatis* infection. As part of a larger effort to estimate *C. trachomatis* infection prevalence at the zonal (collection of districts) level, sample size calculations (assuming 4% prevalence ±2%) called for sampling 1,107 children per zone, which translates to approximately 100 children per district. In each selected cluster, a conjunctival swab was collected from up to 25 present and consented children aged 1–5 years. If two children of this age range were in one household, one child was randomly selected. After trachoma grading, the swab was passed over the conjunctiva three times, rotating 120° between passes.^4,15^ Swabs were placed, dry, into 2-mL vials, placed into coolers with ice packs in the field, and stored in the laboratory at −20°C until they were assayed.

At the Amhara Public Health Institute in Bahir Dar, Ethiopia, swab samples for each of the four districts were randomized, pooled in groups of five individual swabs per pool, and processed with the RealTime (Abbott Molecular Inc., Des Plaines, IL) polymerase chain reaction assay to detect *C. trachomatis* DNA, using the automated Abbott m2000 System.^4^ Laboratory technicians were masked to the district of the sample as well as the trachoma outcomes of the individuals providing the swabs. Laboratory procedures and quality control data have been published previously.^4,16^

### Dried blood spot collection.

Trained laboratory technicians traveling with field teams used a retractable lancet to collect finger prick blood onto a filter paper (TropBio Pty Ltd., Townsville, Queensland) containing six blood spot extensions, each holding approximately 10 µL of blood. Each filter paper was labeled with a bar code, scanned into the survey software, air-dried for at least 2 hours, and then placed into a sealable plastic bag. Filter papers were stored in coolers in the field, then stored at −20°C at the Amhara Public Health Institute.

### Multiplex bead assay.

The DBS were shipped to the CDC in the United States at ambient temperature for testing by serologic assays for antibodies to the *C. trachomatis* antigens Pgp3 and CT694. Antigen-coupled beads were added to 96-well plates (Millipore, Bedford, MA). Control sera and blood spot eluates (1:400) were then added to appropriate wells, beads suspended, plates covered, and shaken at room temperature for 1.5 hours. After washing the beads, total IgG was detected using biotinylated mouse antihuman total IgG (clone H2; Southern Biotech, Birmingham, AL) and biotinylated mouse antihuman IgG4 (clone HP6025; Invitrogen, South San Francisco, CA). After a second wash, streptavidin-phycoerythrin (SAPE Invitrogen) was added at a concentration of 250 ng per well and incubated for 30 minutes at room temperature. Beads were read on a Luminex instrument (Luminex Corp., Austin, TX) equipped with Bio-Plex Manager 6.0 software (Bio-Rad, Hercules, CA). The signal was converted to median fluorescence intensity (MFI) with background levels subtracted out (MFI-BG). Positivity thresholds were generated using a receiver-operating characteristic curve panel of specimens of previously classified positive or negative samples.^5,7^ The positive thresholds for Pgp3 and CT694 were 1558 MFI-BG and 164 MFI-BG, respectively.

### Data analysis.

Reported prevalence estimates for TF were from the whole sample of children aged 1–9 years, were provided by the Tropical Data service, and were calculated using an algorithm which first age-adjusted cluster level data in 1-year age bands using the Ethiopian National Census population. The mean of the cluster prevalence estimates was used to represent the district prevalence.^13^ CIs were calculated using a previously described bootstrap method.^13^ The prevalence of TI in each district was calculated using a similar method to that of TF. As described previously, district *C. trachomatis* infection prevalence was estimated from the district pooled prevalence as the number of positive individual samples most likely to have resulted in observed pooled results.^4,15,17^ Serological outcomes among the serological subsample were described both as continuous variables and as binary variables using the previously described thresholds as cut points. Taylor linearization was used to estimate CIs for serological point estimates to account for the clustered nature of the data using svy procedures in Stata (Stata Corporation, College Station, TX).^12^ Logistic regression was used to test for associations between age and serological markers adjusting for clustering at household and village level. We also estimated *C. trachomatis* force of infection with the seroconversion rate (SCR) to Pgp3 and CT694 among children aged 1–9 years from age-structured seroprevalence using a generalized linear model with a complementary log–log link and robust standard errors.^18,19^ The model assumed stationarity (constant force of infection) and no seroreversion. In sensitivity analysis, we allowed for a range of seroreversion rates between 0.03 and 0.12 per year in a reversible catalytic model, with standard errors estimated using a nonparametric bootstrap that resampled clusters with replacement, stratified by district (1,000 replicates).^5,10^ Analyses were conducted in Stata 13.1 and R version 3.6.1 (R Foundation for Statistical Computing, Vienna, Austria).

## RESULTS

Across the four surveyed districts, a total of 3,429 children aged 1–9 years were examined for trachoma clinical signs, and 442 children from 32 clusters were swabbed for infection. In the serological sample of 78 clusters, 2,391 children aged 1–9 years were examined for trachoma, and among these, antibody response data were available for 2,195 (91.8%) children (Table 1). The age and gender distribution of the serological sample was similar to the distribution of the whole survey sample (Table 2). Improved latrine prevalence was 37.7% for Woreta town, but less than 1% for the other three districts, the prevalence of an improved water source ranged from 22.1% to 94.7%, and the prevalence of water access within 30 minutes ranged between 13.4% and 95.1%.

**Table 1:** Sample sizes for the trachoma impact survey and the serological and infection sub-studies among four districts in Amhara, Ethiopia, 2017

			Full survey		Sub-studies			
Zone	District	Years of surgery, antibiotics, facial cleanliness, and environmental improvement	Clusters, *N*	Total *N*, aged 1–9 years	Serological clusters, *N*	Serological *N*, aged 1–9 years	Infection clusters, *N*	Infection *N*, aged 1–5 years
North Gondar	Alefa	8	30	1,106	22	712	8	120
South Gondar	Woreta town	5	20	532	12	275	8	98
South Gondar	Dera	11	30	967	22	628	8	118
South Gondar	Andabet	11	30	824	22	580	8	106

**Table 2:** Demographic comparisons among the whole trachoma impact survey sample and the serological sample among children aged 1–9 years, and the infection sample among children aged 1–5 years in Amhara, Ethiopia, 2017

Characteristic	Whole sample, *n* (%)	Serological sample, *n* (%)	Infection sample, *n* (%)
Male	1,677 (48.9)	1,055 (48.1)	218 (49.3)
Female	1,751 (51.1)	1,139 (51.9)	224 (50.7)
Age (years)			
1	356 (10.4)	227 (10.3)	95 (21.5)
2	371 (10.8)	244 (11.1)	95 (21.5)
3	346 (10.1)	208 (9.5)	97 (22.0)
4	341 (9.9)	229 (10.4)	80 (18.1)
5	337 (9.8)	216 (9.8)	75 (17.0)
6	371 (10.8)	242 (11.0)	–
7	437 (12.7)	279 (12.7)	–
8	388 (11.3)	236 (10.8)	–
9	482 (14.1)	314 (14.3)	–

### Alefa, previous TF prevalence 4.6%.

**Figure 2.**
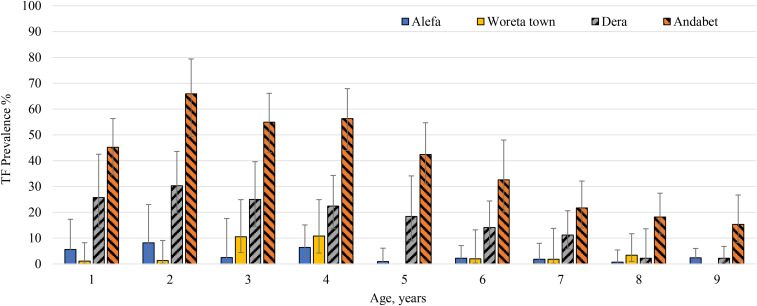
Trachomatous inflammation-follicular (TF) age-specific prevalence among whole district sample (*n* = 3,429) of children aged 1–9 years in four districts in Amhara, Ethiopia. This figure appears in color at www.ajtmh.org.

**Figure 3.**
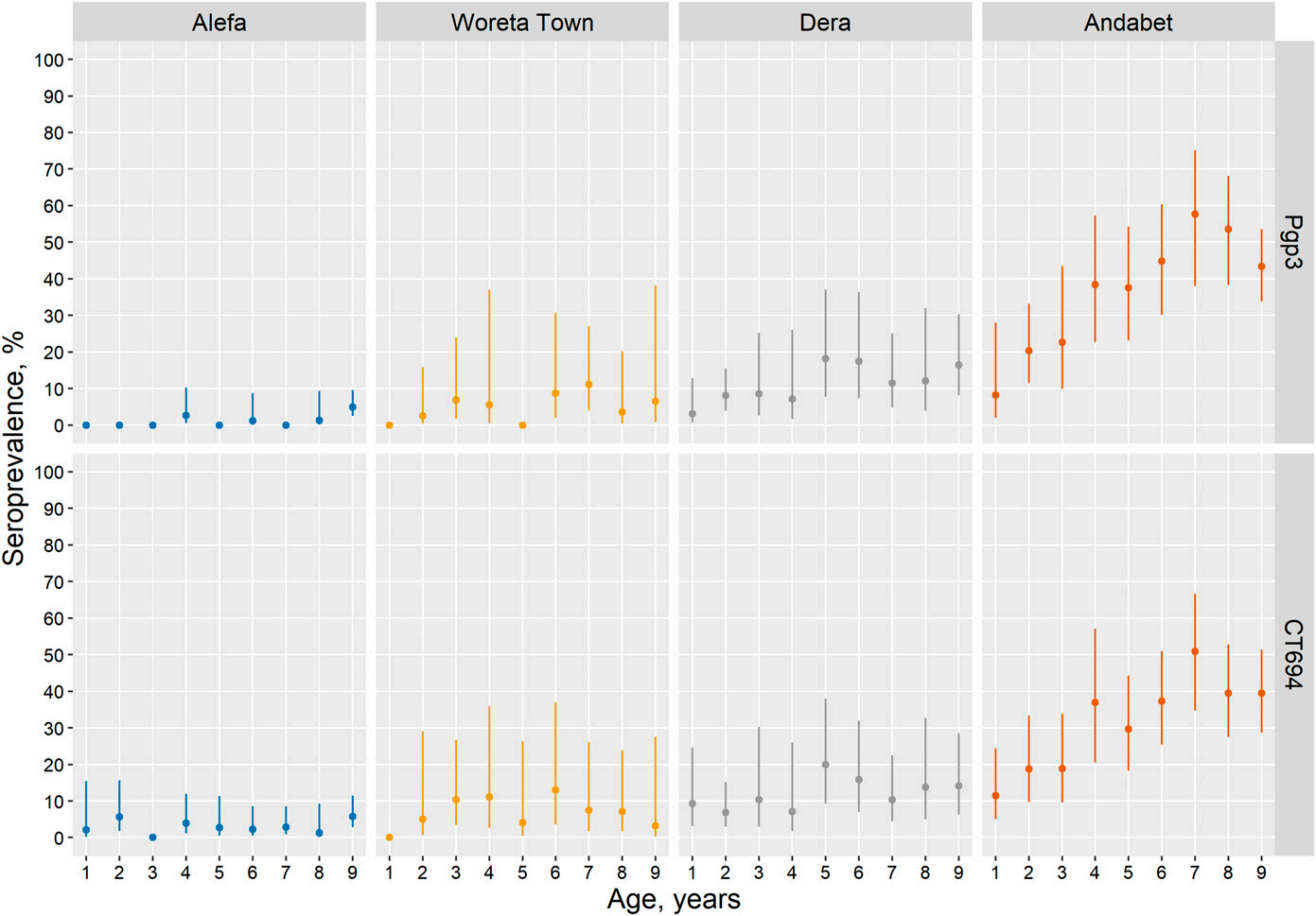
Seroprevalence among children aged 1–9 years (*n* = 2,391) to *Chlamydia trachomatis* Pgp3 and CT694, Amhara, Ethiopia, 2017. Error bars mark 95% CIs. This figure appears in color at www.ajtmh.org.

**Figure 4.**
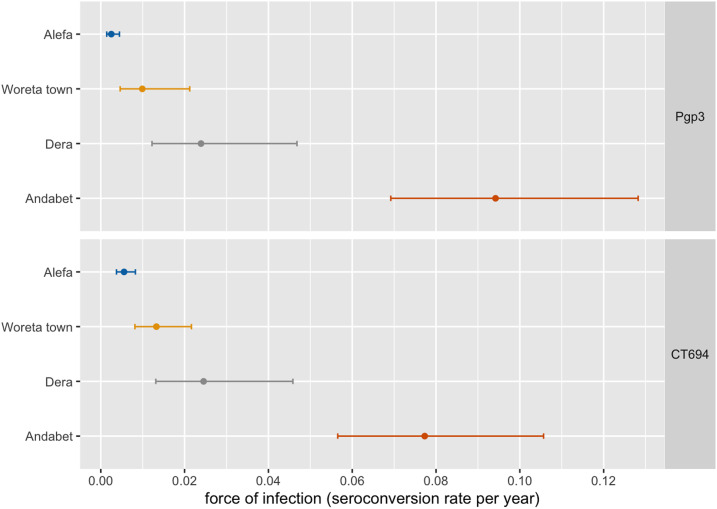
Seroconversion rate per year among children aged 1–9 years, Amhara, Ethiopia, 2017. Error bars mark 95% CIs. This figure appears in color at www.ajtmh.org.

In Alefa, after more than 2 years without MDA, the TF prevalence among children aged 1–9 years remained below the elimination threshold at 3.2% (95% CI: 1.4–5.7) (Table 3). Trachomatous inflammation-intense prevalence was 0.3% (95% CI: 0.0–0.7), and no swabs were positive for *C. trachomatis* infection. Trachomatous inflammation-follicular prevalence did not differ by age (*t* = −2.0, *P* = 0.06) (Figure 2). The prevalence of antibody responses to Pgp3 was 1.4% (95% CI: 0.8–2.5) and to CT694 was 3.1% (95% CI: 2.0–4.6). No children aged 1 year were seropositive for antibodies to Pgp3 in Alefa. Antibodies to Pgp3 showed a statistically significant increase with age (*t* = −2.6, *P* = 0.02), although the highest prevalence observed was 4.9% (Figure 3). In Alefa, the SCR for Pgp3 was 0.003 (95% CI: 0.001–0.004) seroconversions per child-year and the SCR for CT694 was 0.006 (95% CI: 0.004–0.008) (Figure 4).

**Table 3:** District-level prevalence and 95% CIs of trachoma indicators among children aged 1–9 years in the four selected districts of Amhara, Ethiopia, 2017

Zone	District	TF prevalence*	TI prevalence*	Pgp3 prevalence	CT694 prevalence	Pgp3 and CT694 prevalence	Infection prevalence,† %
North Gondar	Alefa	3.2% (1.4–5.7)	0.3% (0.0–0.7)	1.4% (0.8–2.5)	3.1% (2.0–4.6)	1.3% (2.2–4.7)	0
South Gondar	Woreta town	2.7% (1.5–4.5)	0.4% (0.1–0.8)	4.4% (0.2–9.7)	5.8% (3.5–9.6)	2.9% (4.2–12.2)	0
South Gondar	Dera	14.7% (10.0–20.5)	1.3% (0.2–2.8)	11.3% (5.9–20.6)	11.6% (6.3–20.3)	9.7% (7.4–22.5)	0
South Gondar	Andabet	37.0% (31.1–43.4)	6.2% (3.8–8.9)	36.9% (29.0–45.6)	31.9% (24.8–40.0)	29.0% (31.6–48.7)	11.3

TF = trachomatous inflammation-follicular; TI = trachomatous inflammation-intense.

* TF and TI estimates for whole district sample.

† Among children aged 1–5 years; Estimation procedures used do not allow for the calculation of CIs around this point estimate.

### Woreta town, previous TF prevalence 5.4%.

**Figure 5.**
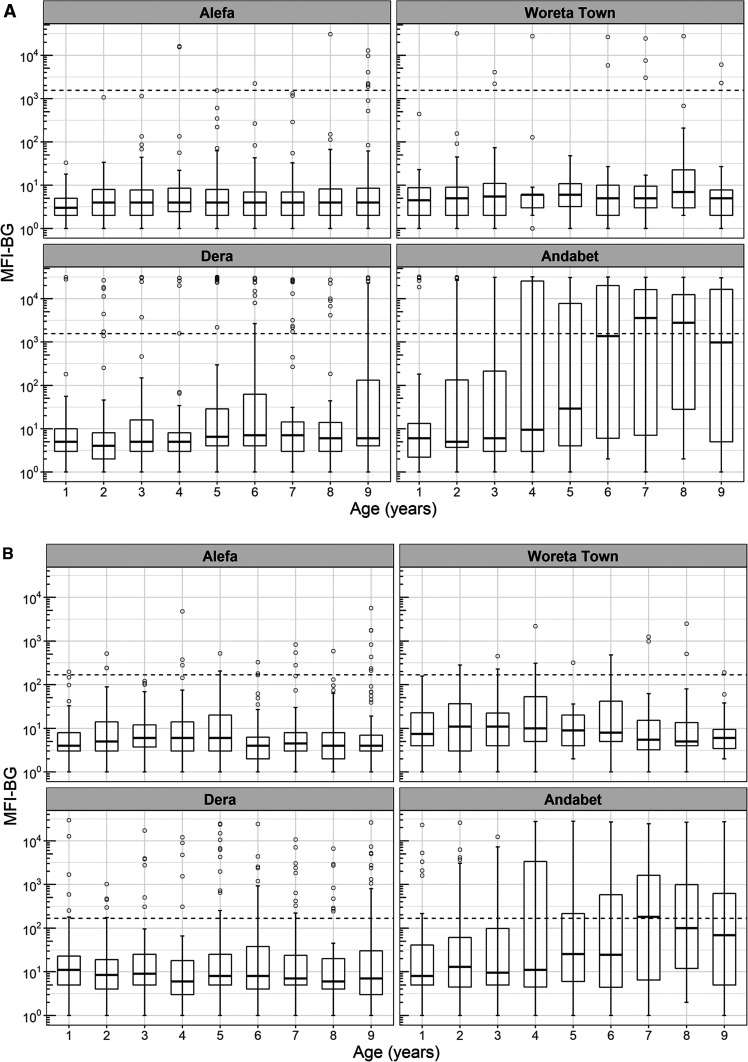
Intensity of anti–*Chlamydia trachomatis* antibody responses among children aged 1–9 years to (**A**) Pgp3 and (**B**) CT694, Amhara, Ethiopia, 2017.

In Woreta town the TF prevalence reached the elimination threshold for the first time, 2.7% (95% CI: 1.5–4.5). The prevalence of TI was 0.4% (95% CI: 0.1–0.8), and no *C. trachomatis* infection was detected. Trachomatous inflammation-follicular prevalence did not differ by age (*t* = −0.3, *P* = 0.75). The prevalence of antibody responses to Pgp3 was 4.4% (95% CI: 0.2–9.7) and to CT694 was 5.8% (95% CI: 3.5–9.6). No children aged 1 year were seropositive for these antibodies in Woreta town. Seroprevalence did not increase with age for either antibody marker for this district. Among individuals seropositive for Pgp3 and CT694, most of the individuals had responses close to the seropositive cut-point (Figure 5A and B). In this district, the SCR for Pgp3 was 0.010 (95% CI: 0.005–0.021) per year, and the SCR for CT694 was 0.013 (95% CI: 0.008–0.022).

### Dera, previous TF prevalence 29.1%.

In Dera, the prevalence of TF was 14.7% (95% CI: 10.0–20.5), and the TI prevalence was 1.3% (95% CI: 0.2–2.8). No *C. trachomatis* infection was detected. Trachomatous inflammation-follicular prevalence was highest in the youngest ages and decreased with age (*t* = −5.1, *P* < 0.001). The prevalence of both antibody markers was less than TF prevalence, Pgp3: 11.3% (95% CI: 5.9–20.6) and CT694: 11.6% (95% CI: 6.3–20.3). The age-related increase in seroprevalence was only statistically significant for Pgp3 (*t* = 2.4, *P* = 0.024), reaching a high of 18.2% among those aged 5 years. In Dera, the SCR per year for Pgp3 and CT694 was 0.024 (95% CI: 0.012–0.047) and 0.025 (95% CI: 0.013–0.046), respectively.

### Andabet, previous TF prevalence 30.6%.

In Andabet district, the TF prevalence remained hyperendemic, 37.0% (95% CI: 31.1–43.4), and the prevalence of TI and *C. trachomatis* infection was 6.2% (95% CI: 3.8–8.9) and 11.3%, respectively. Trachomatous inflammation-follicular prevalence was highest in the youngest ages and decreased with age (*t* = −6.1, *P* < 0.001). The prevalence of antibody responses was 36.9% (95% CI: 29.0–45.6) and 31.9% (95% CI: 24.8–40.0) for Pgp3 and CT694, respectively. Seropositivity increased with age for both markers (Pgp3: *t* = 5.9, *P* < 0.001, CT694: *t* = 4.6, *P* < 0.001) and ranged from 8% in children aged 1 year to more than 50% in those aged 7 and 8 years. The intensity of antibody response was considerably higher in Andabet across the age categories than was observed in the other three districts. The SCR per year for Pgp3 and CT694 in this district was 0.094 (95% CI: 0.069–0.128) and 0.077 (95% CI: 0.057–0.106), respectively. Sensitivity analyses demonstrated that allowing for seroreversion in models would not have a large impact on the SCRs in these districts, particularly at lower levels of transmission (Supplemental Figure 1).

## DISCUSSION

The Amhara region of Ethiopia is historically among the most endemic regions for trachoma in the world, with TF prevalence in children observed as high as 90% in 2003.^20,21^ The Trachoma Control Program in Amhara has documented the enormous effort expended in implementing the A, F, and E arms of the SAFE strategy for trachoma control since scaling up SAFE in 2007, demonstrating acceptable to high MDA coverage using available coverage tools.^12,22,23^ Despite these efforts, many districts in this region have not reached the < 5% TF prevalence threshold after as many as 11 years of SAFE strategy implementation, well in excess of the first estimations of required duration of interventions. The infection and seroprevalence data from these surveys are consistent with the clinical indicators in that the force of *C. trachomatis* infection is still high within a district observed to have TF prevalence ≥ 30% over an 11-year period. The persistent hyperendemic TF prevalence observed in some districts, despite 8–11 years of the SAFE strategy, may reflect a different trachoma epidemiology than what is observed in areas which reach elimination within predicted time lines.^24^

This evaluation included four districts with a range of TF prevalences after multiple rounds of SAFE interventions. Of particular interest was Andabet district, where TF remained ≥ 30% despite 11 rounds of MDA. The high proportion of *C. trachomatis* infection (11.3%), high seroprevalence (36%), and increase in seroprevalence with age (SCR = 0.094) support the clinical indicators that suggest high levels of ongoing trachoma transmission in Andabet district. Under current WHO guidelines, five additional years of SAFE strategy implementations are needed. Although reports from Amhara region have detailed improvements in water, sanitation, and hygiene indicators, it has also been demonstrated that with 50% of households in the region having a latrine and 66% having access to water within 30 minutes, continued improvements are needed.^12^ The existing environmental conditions in Andabet district, as observed in the low prevalence of water and sanitation indicators, are clearly still suitable for *C. trachomatis* transmission. Although low MDA coverage could be partially responsible for the persistent trachoma in Andabet, both administrative and self-reported MDA coverage, from the region as a whole and from highly endemic districts including Andabet, have consistently been close to or greater than the recommended minimum coverage (≥ 80 program coverage) level.^22,23^ Because annual MDA, as part of the SAFE strategy, has yet to reduce the TF prevalence less than 30%, alternatives to annual MDA should be considered for districts such as Andabet experiencing persistent TF. Both modeling studies and recent results from randomized trials have suggested that more frequent MDA or targeted MDA could be viable options.^24–27^ The Trachoma Control Program should consider enhanced MDA strategies while also continuing to focus on the F & E components of the SAFE strategy to reach the elimination threshold faster.

Dera district, which had a previous TF prevalence of 29.1%, was unique in this current assessment in that serological markers were consistently lower than TF prevalence. This is somewhat inconsistent with findings from some studies but not all.^5,28^ It has been assumed that antibody responses are longer lived than TF or *C. trachomatis* infection.^29,30^ A number of studies have demonstrated that TF often overestimates *C. trachomatis* infection in post-MDA settings and can be caused by infections other than *C. trachomatis*.^3,4,31,32^ It is quite possible that despite a TF prevalence higher than 10%, trachoma transmission may have been reduced to low levels in this district. Indeed, no *C. trachomatis* infection was detected in this district, and the Pgp3 SCR was estimated at only 2.4 incident seroconversions/100 children/year. Differences in estimates across trachoma indicators, however, may have been due in part to the different samples used for each indicator. With a current TF prevalence of 14.7% after 11 years of interventions, Dera could also be considered a district experiencing persistent TF.

By contrast, in districts where the TF prevalence was below the threshold for elimination as a public health problem, the prevalence of both serological markers was < 6%, and the seroconversions per 100 children per year were < 1.5, indicating that trachoma transmission was very low.^33^ These data align with survey-based trachoma antibody data from other low-prevalence or post-endemic settings.^5,6,30,34–37^ For example, a recent report of surveillance surveys, conducted in nine Ghanaian districts with low TF, demonstrated a district range of Pgp3 prevalence between 2.5% and 8.2% and 1.3 seroconversions per 100 children per year.^37^ In addition, Pgp3 and CT694 seroprevalence did not increase with age in Woreta town, nor did CT694 seroprevalence increase with age in Alefa, a finding which has been observed in other post-endemic settings.^35,36^ This low seroprevalence in Woreta town and Alefa was most likely due to the lack of *C. trachomatis* exposure. Serological markers may be particularly well suited to detecting the recrudescence of trachoma transmission. Because it is assumed that one or two ocular infections are not enough to develop blinding trachoma, a more cumulative measure of trachoma transmission such as serology would be useful.^38^

This study has several limitations. All districts from which we collected serological data had received at least 5 years of the SAFE strategy, including annual MDA with antibiotics. Although it is difficult to compare quality and quantity of intervention inputs across districts over the long course of the control program, it has been shown that annual MDA coverage has remained high throughout the region, including within these districts.^12,21–23,39^ If adequate population coverage can be assumed, then likely antibody responses reported here were attenuated compared with what would occur if there were no antibiotic pressure on *C. trachomatis* transmission. Indeed, the age–seroprevalence curve observed in hyperendemic Andabet more closely resembled those from areas of meso-endemic (10–30%) trachoma.^8^ Despite the antibiotic pressure, these serological markers still discriminated between districts endemic for trachoma in Amhara and those below the elimination threshold. The infection data as part of these surveys were collected in part to estimate zonal-level infection as opposed to district-level infection.^4^ Given the relatively small number of samples collected, 0% infection should not indicate the complete absence of infection in a district. The district prevalence of serological markers was based on DBS collection from a random selection of 22 of 30 clusters in three districts and 12 of 20 clusters in the fourth, and therefore, it is possible that prevalence may have differed had all clusters been included in the DBS sampling. Although the age and gender distribution of the DBS sample was similar to that of the total sample, future surveys should consider collection of DBS and ocular swabs from all survey clusters, given the community acceptance of this methodology. Last, this serological test cannot discriminate between ocular and genital *C. trachomatis* infection; however, all participants included here were aged ≤ 9 years, reducing the chance of misclassification.

Serological patterns among the key age-group of children aged 1–9 years differed considerably between districts having reached the elimination of trachoma as a public health problem threshold and a district which remains with hyperendemic trachoma. Districts with persistent trachoma such as Andabet should be the focus of increased research around alternative or enhanced antibiotic treatments as well as improved F and E interventions to speed up elimination as a public health problem.

## Supplemental table and figure

Supplemental materials
