# Supplementary material for: Population-Based Prevalence of Chlamydia trachomatis Infection and Antibodies in Four Districts with Varying Levels of Trachoma Endemicity in Amhara, Ethiopia
Source: Am J Trop Med Hyg. 2020 Oct 26;104(1):207–15. doi: 10.4269/ajtmh.20-0777 (PMC7790060; doi:10.4269/ajtmh.20-0777)

Supplemental Figure 1.Seroconversion rate per year among children aged 1 to 9 years assuming various estimated seroreversion rates, Amhara, Ethiopia, 2017. Error bars mark 95% confidence intervals.


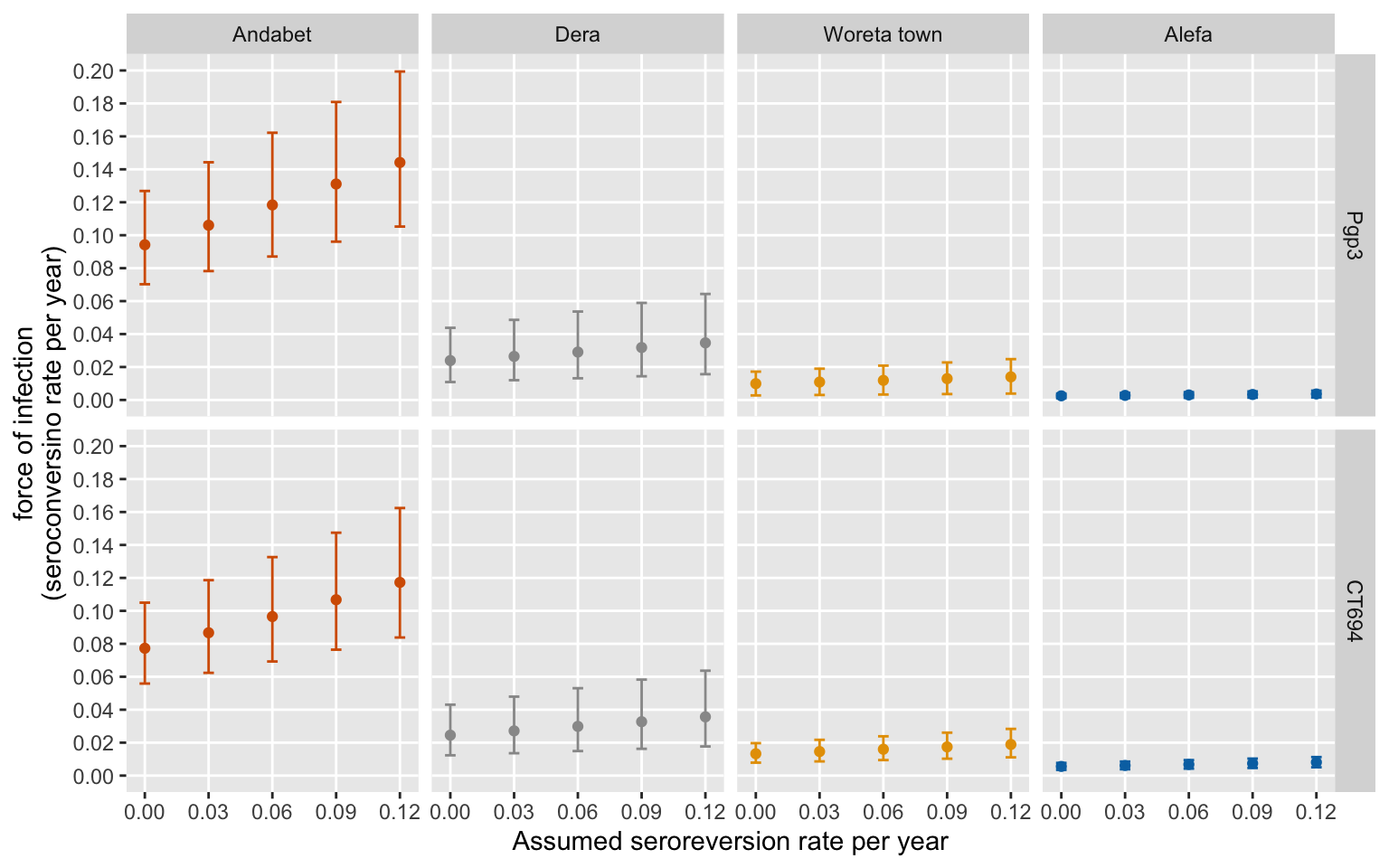

Supplement: Supplementary file 1 [file tpmd200777.SD1.docx]
