# Supplementary material for: Population-Based Prevalence of Chlamydia trachomatis Infection and Antibodies in Four Districts with Varying Levels of Trachoma Endemicity in Amhara, Ethiopia
Source: Am J Trop Med Hyg. 2020 Oct 26;104(1):207–15. doi: 10.4269/ajtmh.20-0777 (PMC7790060; doi:10.4269/ajtmh.20-0777)
Supplement: Supplementary file 2 [file tpmd200777.SD2.docx]

Supplemental Table 1. MDA coverage and prevalence of water and sanitation indicators for the 4 selected districts of Amhara, Ethiopia, 2017.

| Zone | District | MDA Coverage, 2014 | MDA Coverage, 2015 | MDA Coverage, 2016 | Improved Latrine | Improved water source | Time to water < 30 mins |
| --- | --- | --- | --- | --- | --- | --- | --- |
| North Gondar | Alefa | 98.5% | -- | -- | 0.3% | 60.2% | 41.6% |
| South Gondar | Woreta Town | 93.9% | 96.0% | 68.5% | 37.7% | 94.7% | 95.1% |
| South Gondar | Dera | 95.1% | 85.3% | 88.6% | 0.6% | 32.7% | 36.0% |
| South Gondar | Andabet | 92.9% | 93.1% | 92.8% | 0.0% | 22.1% | 13.4% |

*Coverage is determined from administrative reports and defined as the number of doses distributed divided by the target population. MDA=mass drug administration; Improved latrine=pit latrine with slab or pour/flush toilet; Improved water source=protected spring, hand pump/borehole, piped water, rainwater collection; Time to water <30 minutes= time to access bathing or drinking water and return home.
